# *De Novo* Assembly and Developmental Transcriptome Analysis of the Small White Butterfly *Pieris rapae*

**DOI:** 10.1371/journal.pone.0159258

**Published:** 2016-07-18

**Authors:** Lixing Qi, Qi Fang, Lei Zhao, Hao Xia, Yuxun Zhou, Junhua Xiao, Kai Li, Gongyin Ye

**Affiliations:** 1 College of Chemistry, Chemical Engineering, and Biotechnology, Donghua University, Shanghai, 201620, China; 2 State Key Laboratory of Rice Biology, Ministry of Agriculture Key Laboratory of Molecular Biology of Crop Pathogens and Insects, Institute of Insect Sciences, Zhejiang University, Hangzhou, China; National Research Laboratory of Defense Proteins, REPUBLIC OF KOREA

## Abstract

The small white butterfly *Pieris rapae* is one of the most destructive pests of Brassicaceae. Yet little is understood about its genes involved in development. To facilitate research on *P*. *rapae*, we sequenced the transcriptome of *P*. *rapae* during six developmental stages, including the egg, three larval stages, the pupa, and the adult. In total, 240 million high-quality reads were obtained. *De novo* assembly generated 96,069 unigenes with an average length of 1353 nt. Of these, 31,629 unigenes had homologs as determined by a blastx search against the NR database with a cut-off e-value of 10^−5^. Clusters of Orthologous Groups of proteins (COG), Gene Ontology (GO), and Kyoto Encyclopedia of Genes and Genomes (KEGG) analyses were conducted to functionally annotate those genes. Then, 849 genes involved in seven canonical development signaling pathway were identified, including dozens of key genes such as *Hippo*, *Notch*, and *JAK2*. A total of 21,883 differentially expressed (cut-off of 2-fold) unigenes were detected across the developmental stages, most of which were found between the egg and first larval stages. Interestingly, only 34 differentially expressed unigenes, most of which are cuticle protein related genes, were detected with a cut-off of 2^10^-fold. Furthermore, we identified 32 heat shock protein (Hsp) genes that were expressed with complete open reading frames. Based on phylogenetic trees of the *Hsp* genes, we found that *Hsp* genes with close evolutionary relationships had similar expression pattern. Additionally, partial pattern recognition receptors genes were found to be developmental regulated. This study provides comprehensive sequence resources for *P*. *rapae* and numerous differential expressed genes, and these findings will lay the foundation for future functional genomics studies on this species.

## Introduction

The cabbage butterfly, *Pieris rapae* (Lepidoptera: Pieridae) is one of the most destructive agricultural pests in the Brassicaceae family, causing considerable economic loss in China [1]. *P*. *rapae* and its natural enemy *Pteromalus puparum* provide an important physiological interaction model, for the study of innate immunity. Dozens of immunity-related genes have been cloned, including the serotonin receptor [2], the ryanodine receptor [3], and prophenoloxidase [4]. Although the mitochondrial genome has been sequenced [5], the nuclear genome remains unavailable. Additionally, functional transcriptome information may lead to a global vision of gene expression, a profound understanding of the biology of this insect, as well as the development of integrated control strategies.

In Lepidoptera, the genome sequences of more than five species and transcriptomes of dozens of species [6] have been sequenced, while only some have developmental transcriptomes available. A comparison of the developmental transcriptome of insects from egg to adult would provide insights into the function of numerous genes and the regulation of different signaling pathways involved in different developmental stages [7–11]. For example, most of the differentially expressed genes were found in Lepidopteran *Athetis lepigone* egg and larva [11]. The most differentially expressed genes were involved in metabolic pathways [12]. In addition, knowledge of changes in immunity-related genes in different developmental stages may provide biologically important information about immune function during development [13].

We attempted to obtain expression data throughout all developmental stages, by performing comprehensive RNA sequencing on *P*. *rapae*. We hoped to obtain high quality assembled transcripts and to discover development related genes during various periods of insect growth by performing *de novo* transcriptome assembly. The result produced a high-quality reference transcript set for future research on this species.

## Materials and Methods

### Ethics statement

There are no specific permits required for collection of *P*. *rapae*, which is not a protected or endangered insect species. There are no ethical issues involved in this research.

### Insects

*P*. *rapae* larvae were collected from Shanghai, China in May 2014 and reared at 25 ± 1°C, a relative humidity of 80 ± 5%, and a photoperiod of 10D:14L on cabbage leaves until they developed into pupae. Pupae were maintained in the same conditions as described above until emergence. Samples were collected and washed twice in ddH_2_O in filter paper. Clean samples were reserved in RNAlater (Qiagen, Germany) and stored at -20°C until RNA extraction. In total, 40 eggs, six larvae six pupae and three pairs of adults were used as sequencing samples.

### cDNA Synthesis and sequencing

Total RNA was isolated from the samples at six developmental stages of *P*. *rapae* using an RNeasy MinElute Cleanup Kit (Qiagen, Germany) according to the manufacturer's instructions. The RNA concentration was assessed using a Qubit fluorometer (Invitrogen Corp. USA) and the quality was confirmed using a 2100 Bioanalyzer (Agilent Technologies, USA). Stage-specific samples from RNA extractions were pooled at an equal concentration. cDNA libraries were constructed using a TruSeq^TM^ RNA Sample Preparation Kit v2 according to the manual (illumina®, San Diego, CA, USA). Briefly, the poly-A containing mRNA molecules were purified using poly-T oligo-attached magnetic beads with two rounds of purification. During the second elution of the poly-A RNA, the RNA was also fragmented and primed for the first-strand cDNA synthesis. The RNA templates were removed and the second-strand cDNA was synthesized to generate double-stranded (ds) cDNA. Ds cDNA ends were repaired and a single ‘A’ nucleotide was added to the 3’ ends of the fragment. Then, the adapters were ligated to the end of the ds cDNA, and prepared for hybridization onto a flow cell. Finally, PCR was use to selectively enrich DNA fragments with adapter molecules on both ends and to amplify the amount of DNA in the library. The libraries were sequenced on an Illumina GAIIx (illumina®, San Diego, CA, USA) platform with sequence runs of 2 × 125 bp paired-ends at the laboratory of WuXi AppTec (Shanghai, China).

### Sequence assembly

We performed a rigid filtering process with a cut-off Q-value of 30 using the NGS QC Toolkit [14]. High-quality reads had a Phred score over 30 across more than 70% of the bases. These high quality reads were prepared for *de novo* transcriptome assembly using Trinity software [15, 16], with default parameters. Briefly, Trinity combined certain lengths of overlapping reads and paired-end information to assemble longer fragments, which then generated transcripts and components. These components were termed unigenes, which were submitted to the subsequent annotation analysis.

### Functional annotation

Unigenes were first blasted against the NCBI non-redundant (NR) protein database using Blastx with an e-value cutoff of 1E-5. The best blast hits were used to describe the features of unigenes, such as the gene name, species homology, and identity distribution. Next, blast results were functionally annotated with Gene Ontology (GO) terms using Blast2GO software [17] and the GO functional classification for all of the annotated unigenes was conducted using WEGO software [18]. In addition, Clusters of orthologous groups of proteins (COG) and Kyoto Encyclopedia of Genes and Genomes (KEGG) annotations were performed to further explore the unigenes (http://weizhongli-lab.org/metagenomic-analysis/server/). Unigenes were translated into amino acid sequences by TransDecoder software, which is part of the Trinity software [15, 16].

### Differential gene expression analysis

To obtain gene expression information, we calculated abundance estimation for each sample against assembly sequences using RSEM software [19]. Then, the expected counts of all samples were gathered and edgeR software [20] was used to compare the gene expression across samples. The value of log_2_^(fold change)^ was used to compare two stages. Three criteria were used to describe differential gene expression with a false discovery rate (FDR) less than 0.001. First, log_2_^(fold change)^ > 2 between any adjacent stages was used to identify significantly differential expression. Second, log_2_^(fold change)^ > 2 between all of the adjacent stages was used to identify genes with frequently changed expression levels. Finally, genes expressed in unique stages were identified as stage-specific unigenes.

### Reconstruction of phylogenetic trees

Mega 5.0 software [21] was used to construct consensus phylogenetic trees using the neighbor-joining method. Branch strength was evaluated by a bootstrap analysis of 500 replication trees.

## Results

### Sequencing and quality filtering

In total, approximately 282 million paired 125 bp reads were obtained from six developmental stages of *P*. *rapae* (S1 Table) using HiSeq2000. Within each stage, there were more than 42 million raw reads. All raw reads were submitted to the NCBI Sequence Read Archive under accession numbers SRX336675 and SRR954044 associated with BioProject accession number PRJNA215794 and BioSample accession number SAMN02319001. After quality filtering and removing low quality-reads, approximately 240 million high-quality reads (87.45%) remained and were pooled together for assembly.

### *De novo* assembly characteristics

**Fig 1:**
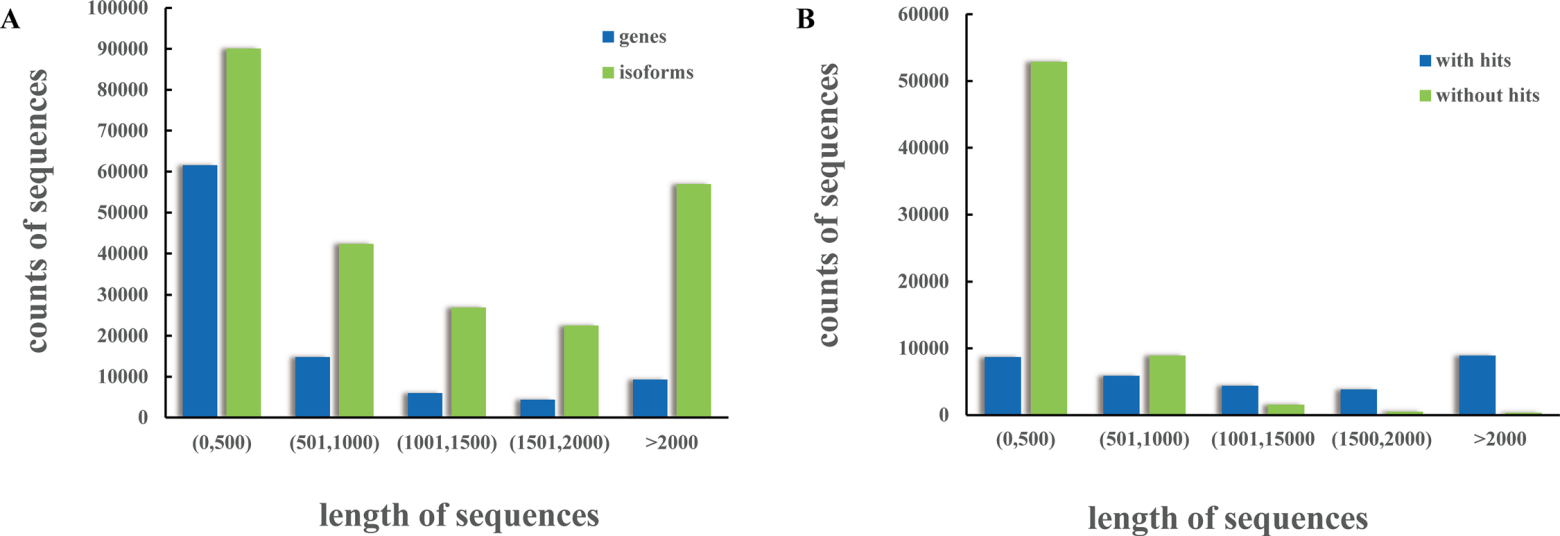
Length distribution of assembled sequences. (A) The counts of genes and isoforms assembled decreases along with length. The length of genes and isoforms are represented on the X-axis, while the number is on the Y-axis. (B) The counts of unigenes without matches (with a cut-off E-value of 10E-5) in the NCBI NR database decreases along with length. The length of matched and unmatched genes are represented on the X-axis while the number of them is on the Y-axis.

Using the Trinity assembly program [15, 16], the *P*. *rapae* transcriptome was reconstructed with high-quality (HQ) reads pooled from all stages. The assembly generated 238,738 transcripts with an N50 value of 2471 nt and 96,069 unigenes with an average length of 1, 353 nt. Reads libraries from each stage were also assembled separately, resulting in lower numbers of unigenes in larva and higher number in adults (Table 1). The GC contents of the *P*. *rapae* transcriptome were approximately 40% across all stages, which was higher than that of *Bombyx*. *mori* (37.7%) and *Microplitis demolitor* (30.4%). The distribution of unigenes and transcripts was analyzed in a 500 nt window in Fig 1A.

**Table 1:** Summary for *Pieris rapae* developmental transcriptome.

Stage	transcripts			unigenes			Total assembled bases
	Number	GC%	N50	number	Median	Average Unigene	
E	101,274	38.74	2,487	22703	854	1,444.43	146,283,171
L1	105,228	40.61	2,100	49,165	803	1,251.53	131,695,912
L3	63,204	40.88	1,321	43,807	479	816.43	51,601,445
L5	63,535	39.99	1,374	42,187	489	848.17	53,888,489
P	92,776	40.28	2,104	44,671	811	1,255.81	116,508,688
A	104,668	40.39	2,303	47,717	905	1,370.61	143,458,521
ALL	238,783	39	2471	96,069	798	1,353.27	330,715,242

E, L1, L3, L5, L, P, and A refer to egg, first stage of larva, third stage of larva, fifth stage of larva, pupa, and adults of *P*. *pieris*, respectively. The unit of N50, Median, and Average Unigene is nt (nucleotides).

### Functional annotation and classification

**Fig 2:**
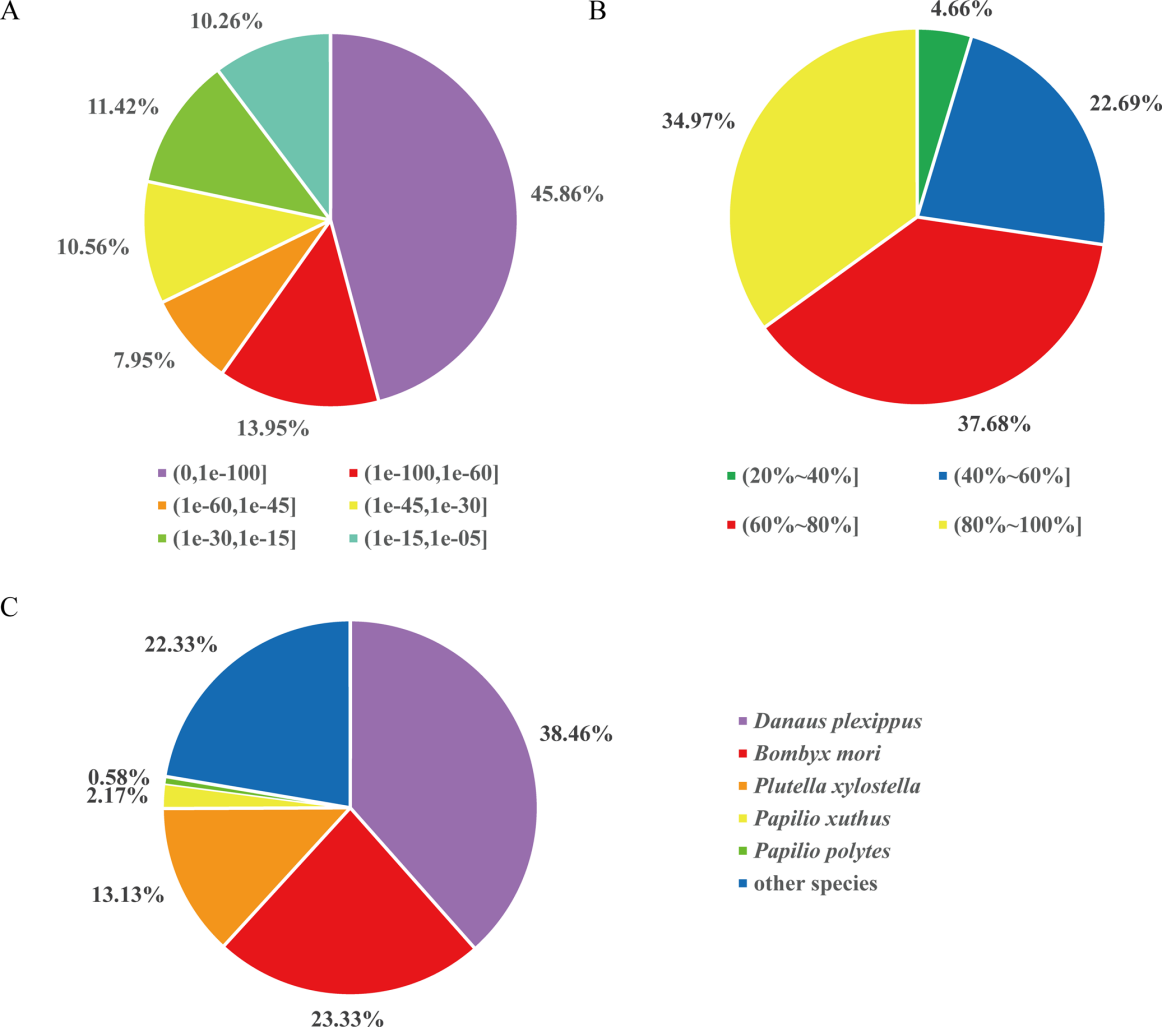
Characteristics of homology search of genes against the NR database. (A) E-value distribution of BLAST hits for each unigene with a cut-off of E-value of 10E-5. (B) Similarity distribution of the top BLAST hits for each unigene. (C) Species distribution is shown as percentage of the total homologous gene hits. All of the top five species are Lepidoptera.

A homology analysis of the *P*. *rapae* unigenes was performed using BlastX against non-redundant databases. Longer unigenes tend to have more homologs compared to short ones (Fig 1B). In total, approximately 33% (31,629) of the *P*. *rapae* unigenes were found to be homologs in the NR database with an e-value smaller than the cutoff. A comparative analysis of the e-value distribution of hit unigenes shows that 45.86% of *P*. *rapae* unigenes have the highest homology with an e-value cut-off smaller than 1E-100 (Fig 2A). Likewise, in the similarity distribution of hit unigenes (Fig 2B), over 75% of them had more than 60% similarity to homologs. As expected, the top unigene hit were found in insect genomes, especially Lepidoptera. *Danaus plexippus* (38.4%), *B*. *mori* (23.3%), *Plutella xylostella* (13.1%), *P*. *xuthus* (2.2%), and *Papilio polytes* (0.6%), had the top five counts of unigenes with NR annotation (Fig 2C).

**Fig 3:**
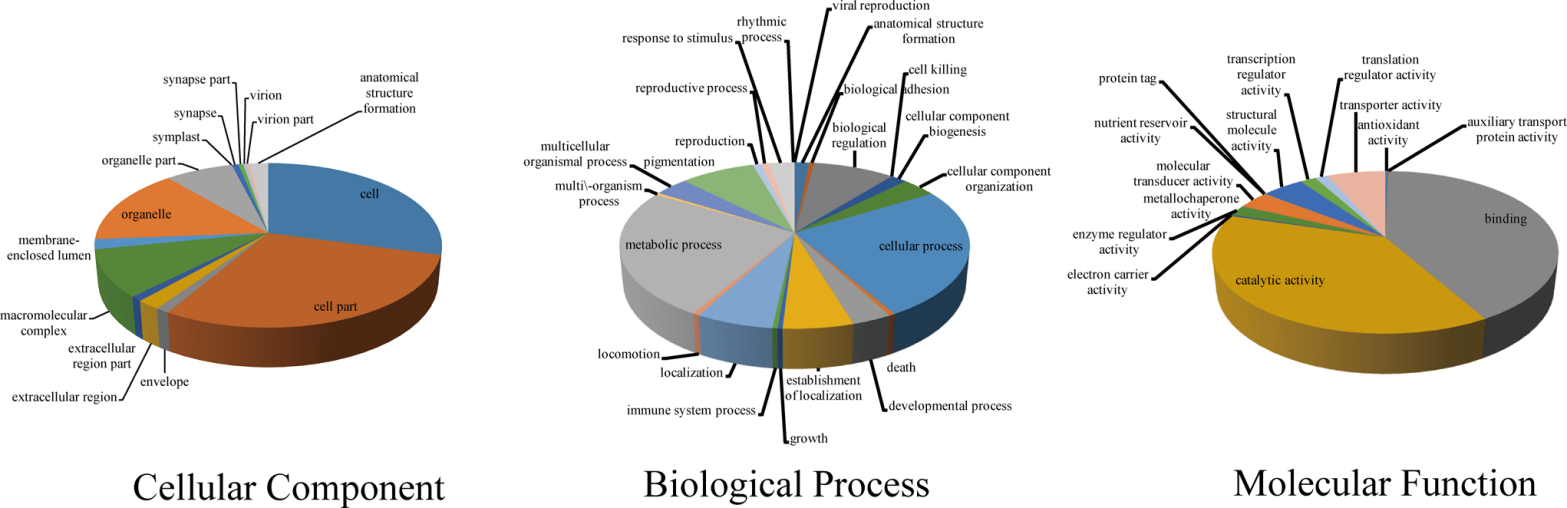
Gene ontology (GO) assignment for the Pieris rapae transcriptome. GO assignments for predicted for their involvement in pie charts (A) biological processes, (B) cellular components, and (C) molecular function.

The GO (gene ontology) database (http://geneontology.org/), developed by the Gene Ontology Consortium, was used for standard gene function annotation, providing a dynamic, controlled vocabulary that can be applied to multiple species [22]. Using Blast2Go software, the transcriptome of *P*. *rapae* was successfully mapped to three main functional processes, which were composed of 51 GO terms (Fig 3). Based on the GO analysis, approximately 43% of the genes were classified as related to biological processes. The remaining genes were classified as related to cellular processes (33%) and molecular processes (24%).

**Fig 4:**
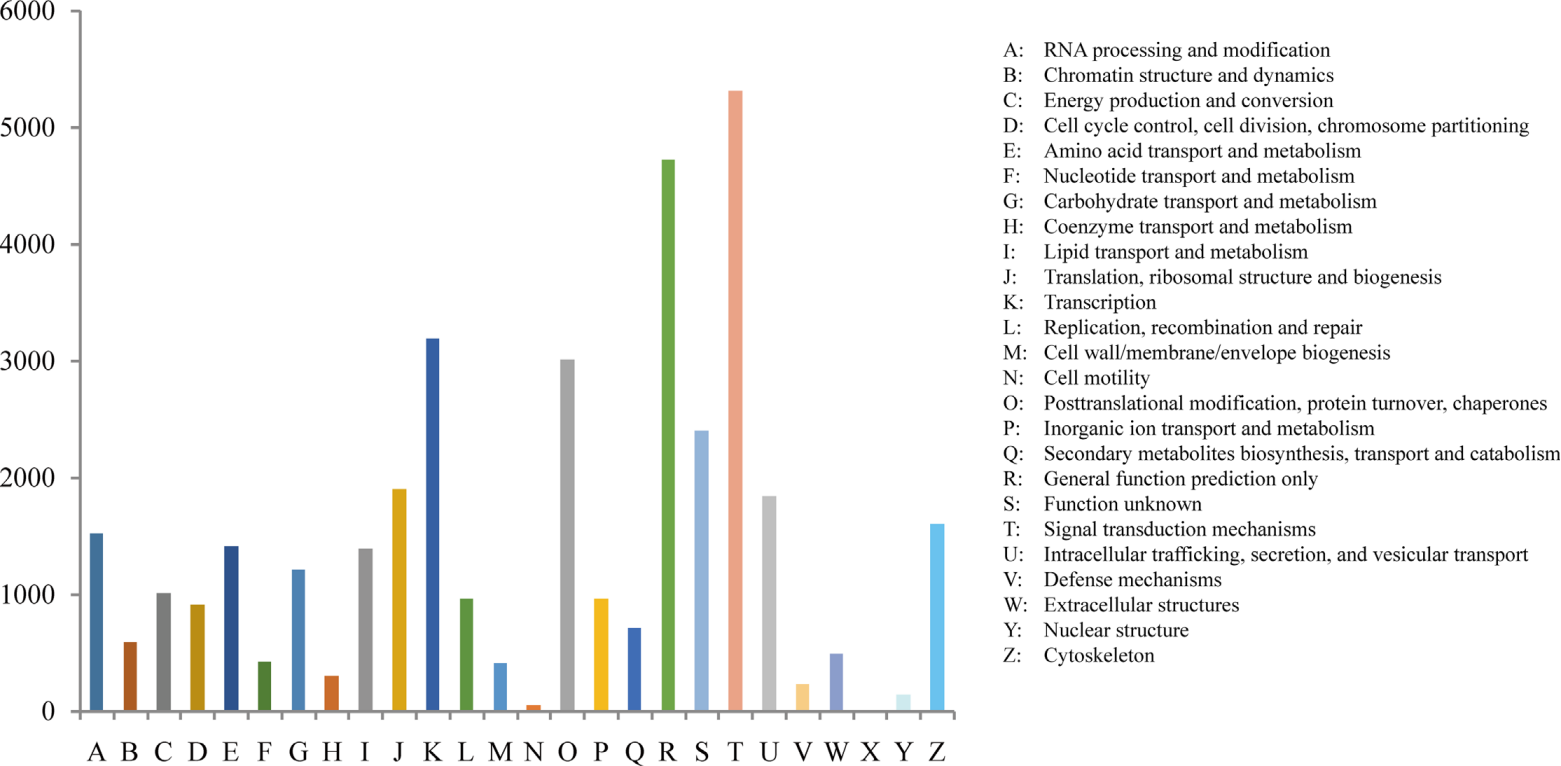
Histogram presentation of clusters of orthologous groups (KOG) classification. The X- axis is the KOG term. The Y-axis shows the number of unigenes.

Clusters of orthologous groups of proteins (COGs) represent a phylogenetic classification of the protein database involving eukaryotes (KOG) and prokaryotes (COG) [23, 24]. Before COGs classification, we transformed the RNA sequences of unigenes into protein sequences with TransDecoder software, which is a part of Trinity [16]. A total of 17,323 proteins were assigned to 1369 COG terms, which were classified into 25 COG groups, and 32,830 proteins were assigned to 43,48 KOG terms, which were classified into 26 KOG groups with an e-value cut-off of 1E-5 (Fig 4) using WebMGA [25]. In the KOG classification, the largest group was “signal transduction mechanisms”, followed by “general function prediction only”, “transcription”, “posttranslational modification, protein turnover, chaperones”, and “translation, ribosomal structure and biogenesis” (Fig 4). In the COG classification, the largest group was “general function prediction only”, followed by “posttranslational modification, protein turnover, chaperones”, “translation, ribosomal structure and biogenesis”, “transcription”, and “replication, recombination and repair” (Fig 4).

Based on the KEGG pathway mapping analysis and annotation of all unigenes, 13,225 unigenes were successfully mapped to 335 KEGG pathways. There were 46 (13.7%) pathways involving more than 300 unigenes. The top three pathways were as follows: metabolic pathways (3620 genes), biosynthesis of secondary metabolites (1214 genes), and biosynthesis of antibiotics (789 genes). Considerable numbers of unigenes were involved in the development related pathway of *P*. *rapae*, and approximately 849 unigenes were associated with seven signaling pathways. The largest signaling pathway was MAPK (231 genes), followed by Wnt, Fox, Hippo, Hedgehog, and Jak-STAT (Table 2 and S2 Table). Some main nodes of these identified signaling pathways identified are also listed in Table 2. Additionally, the key genes involved in the innate immunity were identified including the Toll and IMD (immune deficiency) pathways as well as pattern recognition receptors (S3 Table and S1 File).

**Table 2:** Development related pathways and partially annotated key genes.

KEGG pathway	Unigene number	Partial unigene annotation
Jak-STAT signaling pathway	51	JAK2, STAT, STAM, PI3K, SHP2,CBL, SHP1
Wnt signaling pathway	158	WNT1, WNT4, WNT5, WNT6, WNT7, WNT10, WIF1, frizzled, sinah, arm, dsh
Notch signaling pathway	46	Notch, Su(H), delta, deltex, CIR, hairles
Hedgehog signaling pathway	60	Hedgehog, gas1, PTCH1, SMO, Fu, CK1, PKA, Slimb
Hippo signaling pathway	181	Hippo, yki, wts, mats, Gug, Mad, sd,
MAPK signaling pathway	231	EGFR,GRB2, Sos, ras, phl, Dsor1, rl
FoxO signaling pathway	122	FoxO3, FoxG, atg12,

### Gene expression and developmental stages analysis

**Fig 5:**
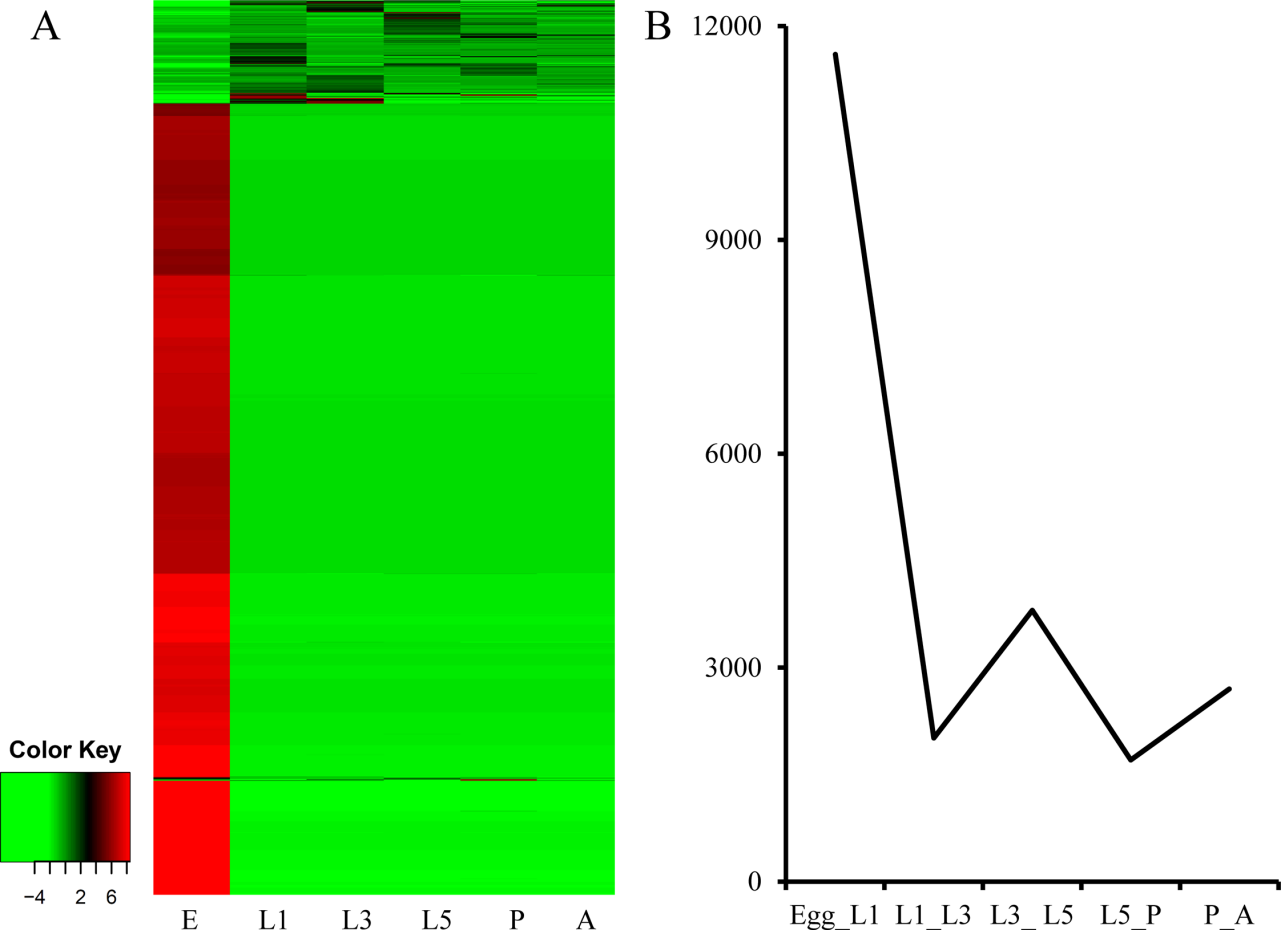
A heat map of genes expression and numbers of genes that significantly change in the *Pieris rapae* transcriptome druing development. E, L1, L3, L5, L, P, and A refer to the egg, the first stage of larva, the third stage of larva, the fifth stage of larva, the pupa, and the adult of *P*. *rapae*, respectively. (A) A heat map for the average transformed numbers of RPKM values of genes in the *P*. *rapae* transcriptome. (B) Numbers of genes with 2-fold changed in adjacent stages during development.

RSEM [19] was used to estimate the abundance of different genes to investigate the global transcriptional differences across stages during development. In total, 21,883 transcripts were found to be significantly differentially expressed between any pair of developmental stages with p-value less than 10E-3. Approximately, 53% of differentially expressed transcripts had a higher expression during the egg stage (Fig 5A), which is consistent with the expression pattern of *D*. *melanogaster* [26]. The top five pathways in the egg stage were cancer (582 genes), focal adhesion (536 genes), the regulation of the actin cytoskeleton (506 genes), RNA transport (468 genes), and purine metabolism (430 genes). Furthermore, the number of differentially expressed genes decreased after the egg stage (Fig 5B).

To identify transcripts that were uniquely expressed in the egg, L1, L3, L5, pupae, or the adult stages, we used custom scripts to filter transcripts expressed in any two stages. A total of 9724 transcripts were found to be stage-specific. Among them, most stage-specific transcripts were found in the egg stage (Table 3 and S4 Table). Partial stage-specific unigenes are listed in Table 3, and all annotated stage-specific transcripts are listed in S4 Table.

**Table 3:** Number of the stage specific expressed genes over development.

	nunber	Blast hit	partial annotated genes
E	9560	4439	SOX2, TP73, eiger, RASSF2_4, MP1, PTPRR
L1	59	10	HRG
L3	10	3	SETMAR, desaturase
L5	14	3	uncharacterized
P	7	1	tetrameric potassium-selective cyclic nucleotide gated channel
A	74	37	Opsin, mucin, abp3, gobp2, ShxC

E, L1, L3, L5, L, P, and A refer to egg, first stage of larva, third stage of larva, fifth stage of larva, pupa, and adults of *P*. *pieris*, respectively.Abp3: antennal binding protein 3; gobp2: general odorant-binding protein 2, uncharacterized, Hrg: histidine-rich glycoprotein, TPSCNGC, tetrameric potassium-selective cyclic nucleotide gated channel.

To further investigate the frequently changed gene expression level, we found 39 transcripts that were significantly differentially expressed at all pairwise adjacent developmental stages. Then, 33 of them were annotated (Table 4). Interestingly, 19 of 33 transcripts were annotated to the cuticular protein genes of Lepidoptera insects, such as *D*. *plexippus*, *B*. *mori*, *P*. *polytes*, *Biston betularia*, and *P*. *xuthus*. One of 33 was predicted to be arylalkylamine N-acetyltransferase, which is involved in the day/night rhythmic production of melatonin. The rest of the annotated genes are uncharacterized.

**Table 4:** Differential expression genes at all pairwise adjacent developmental stages.

uigene_ID	Annotation description
comp90916_c0_seq1	arylalkylamine N-acetyltransferase
comp100119_c0_seq1	cuticular protein CPFL4A
comp99705_c0_seq1	cuticular protein CPFL4A
comp93091_c0_seq1	cuticular protein glycine-rich 13
comp93091_c1_seq1	cuticular protein glycine-rich 13
comp107702_c1_seq1	cuticular protein PpolCPG12
comp95222_c0_seq1	cuticular protein PpolCPH30
comp95086_c0_seq1	cuticular protein PpolCPH30
comp92039_c0_seq1	cuticular protein PxutCPR6
comp92026_c0_seq1	cuticular protein PxutCPR6
comp95785_c0_seq1	cuticular protein PxutCPR91
comp99242_c0_seq1	cuticular protein RR-1 motif 17
comp99239_c0_seq1	cuticular protein RR-1 motif 17
comp101300_c0_seq1	cuticular protein RR-2 family member 59 precursor
comp96531_c1_seq1	cuticular protein tweedle motif 1
comp103412_c0_seq1	hypothetical protein KGM_00533
comp104056_c0_seq1	hypothetical protein KGM_00533
comp92032_c0_seq1	hypothetical protein KGM_07587
comp92032_c1_seq1	hypothetical protein KGM_07587
comp114300_c0_seq1	hypothetical protein KGM_08048
comp103785_c0_seq1	hypothetical protein KGM_08420
comp104376_c0_seq1	hypothetical protein KGM_08420
comp105155_c0_seq1	hypothetical protein KGM_15892
comp105279_c0_seq1	hypothetical protein KGM_15892
comp106145_c0_seq1	hypothetical protein KGM_21191
comp108475_c0_seq1	LOC101744689 isoform X1
comp108630_c0_seq1	LOC101744689 isoform X1
comp105225_c0_seq1	Collagen alpha-1V chain
comp107452_c0_seq1	TPA: putative cuticle protein
comp98894_c0_seq1	TPA: putative cuticle protein
comp93261_c0_seq1	cuticular protein CPFL family 3 precursor
comp92650_c0_seq1	cuticular protein CPFL family 3 precursor
comp105018_c1_seq1	cuticular protein RR-2 motif 102 precursor
comp97362_c0_seq1	cuticular protein RR-2 motif 98 precursor

### Heat shock protein (HSP) genes analysis

After searching the automated database and a careful manual review, a total of 32 *Hsp* genes with complete open reading frames in *P*. *rapae* were identified as members of six *Hsp* families, including *sHsp*, *Hsp10*, *Hsp40*, *Hsp60*, *Hsp70*, and *Hsp90*. All of the best blast hits were found in Lepidoptera. The numbers of identified genes in the *sHsp*, *Hsp10*, *Hsp40*, *Hsp60*, *Hsp70*, and *Hsp90* families of *P*. *rapae* were 17, 1, 3, 2, 5 and 4, respectively. Detailed information about *Hsp* genes in *P*. *rapae* including their unigene IDs and the deduced amino acid sequences is listed in Table 5 and S1 File. Hsp10, Hsp60 and Hsp70 proteins displayed high similarity to their counterparts in other organisms, followed by Hsp90. sHsp showed lower similarities to insect small Hsps of other insects.

**Table 5:** Putative identified heat shock protein genes with complete open reading frame.

Family	Unigene ID	Length	Subject id	Identity	Hit Species	MW
Hsp10	comp87897_c0	658	XP_004932946.1	91.26	*Bombyx mori*	11.25
Hsp40	comp104355_c0	1608	NP_001040115.1	56.41	*B*. *mori*	27.08
	comp103272_c0	1571	NP_001040115.1	56.41	*B*. *mori*	27.09
	comp93707_c0	1419	BAM18277.1	90.65	*Papilio xuthus*	39.99
Hsp60	comp97942_c0	2200	ACT52824.1	95.69	*Chilo suppressalis*	60.99
	comp99373_c0	2358	ACT52824.1	96.21	*C*. *suppressalis*	60.99
hsp70	comp103665_c0	2567	XP_011555994.1	95.11	*Plutella xylostella*	72.62
	comp97017_c0	2188	AIA61347.1	99.84	*Pieris rapae*	71.67
	comp103497_c0	2183	EHJ73891.1	92.54	*Danaus plexippus*	68.91
	comp115029_c0	3156	AET10425.1	99.1	*P*. *rapae*	75.5
	comp107622_c0	2001	AGR84218.1	79.02	*Melitaea cinxia*	68.71
hsp90	comp105881_c0	2703	NP_001266361.1	84.4	*B*. *mori*	77.13
	comp105326_c0	2673	NP_001266361.1	84.56	*B*. *mori*	77.12
	comp97149_c0	2537	AET10426.1	98.19	*P*. *rapae*	82.71
	comp97350_c0	2878	ADK55517.2	80.03	*Spodoptera litura*	89.14
sHsp	comp90845_c0	760	AAZ14792.1	83.15	*Choristoneura fumiferana*	19.79
	comp101795_c0	1185	EHJ77259.1	64.8	*Danaus plexippus*	26.81
	comp100208_c0	3258	AET10424.1	100	*P*. *rapae*	19.9
	comp101221_c0	888	EHJ73481.1	36.77	*D*. *plexippus*	17.46
	comp99691_c0	871	EHJ73481.1	36.13	*D*. *plexippus*	17.46
	comp91410_c0	731	XP_004927349.1	81.44	*B*. *mori*	19.46
	comp92450_c0	726	XP_004927349.1	81.44	*B*. *mori*	19.54
	comp100377_c0	1366	ABS57447.1	97.33	*Heliconius erato*	21.4
	comp84890_c0	995	EHJ70499.1	63.54	*D*. *plexippus*	22.69
	comp78900_c0	574	EHJ71411.1	50.91	*D*. *plexippus*	19.37
	comp103102_c0	1270	ADK55523.1	36.22	*S*. *litura*	33.92
	comp103335_c0	1218	ADK55523.1	38.26	*S*. *litura*	19.16
	comp97707_c0	773	AHW45922.1	78.03	*P*. *xylostella*	20.88
	comp97900_c0	776	AHW45922.1	78.74	*P*. *xylostella*	20.9
	comp93345_c0	852	ADK55523.1	44.86	*S*. *litura*	24.17
	comp96843_c0	873	EHJ77277.1	77.78	*D*. *plexippus*	20.05
	comp90287_c0	773	EHJ74663.1	73.02	*D*. *plexippus*	21.34

Identity, sequence identity to subject protein (%); MW, predicted molecular weight (kDa); Length, length of unigenes in *P*. *rapae*; Species, blast hit organism.

**Fig 6:**
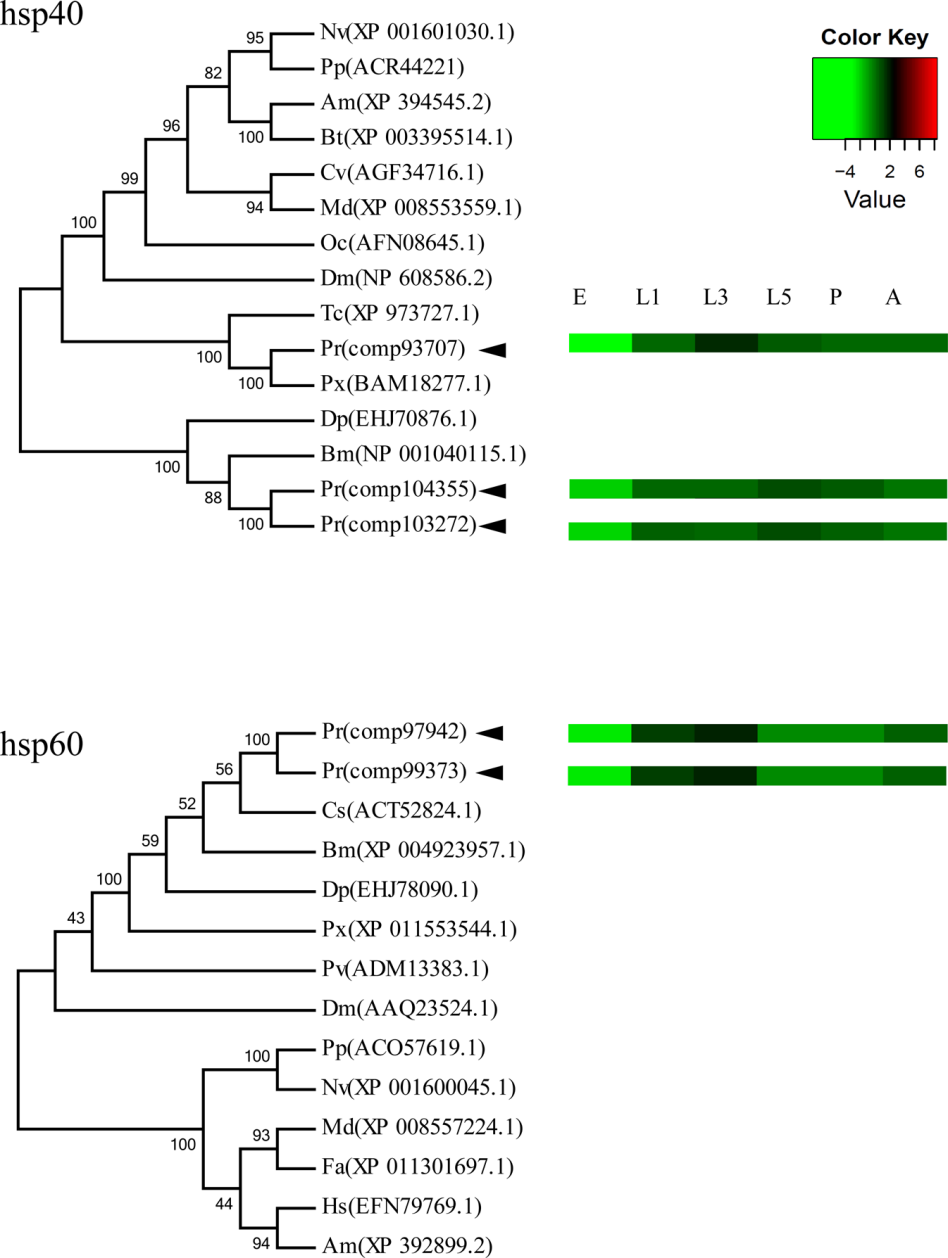
The homology relationships of *Pieris rapae* heat shcok proteins with their counterparts in other insect species as well as expression levels in *P*. *rapae* during development. The trees were generated using the NJ method in Mega5 software. Expression levels were compared with their RPKM values. The other Hsp amino acid sequences used are from Nv (*Nasonia vitripen*nis), Am (*Apis mellifera*), Bt (*Bombus terrestris*), Cv (*Cotesia vestalis*), Md (*Microplitis demolitor*), Oc (*Oxya chinensis*), Dm (*Drosophila melanogaster*), Tc (*Tribolium castaneum*), Pr (*Pieris rapae*), Px (*Papilio xuthus*), Dp (*Danaus plexippus*), Bm (*Bombyx mori*), Cs (*Chilo suppressalis*), Pv (*Polypedilum vanderplanki*), Fa (*Fopius arisanus*), and Hs (*Harpegnathos saltator*).

To explore the evolutionary relationship of the Hsp proteins, a phylogenetic analysis of each family was performed based on full-length amino acid sequences from *P*. *rapae* (Fig 6 and S2 File). The Hsp70 family could be classified into the following two subfamilies: heat shock cognate protein 70 and heat shock inducible 70. Interestingly, when we compared the expression levels and phylogenetic relationships, we found that unigenes with closer evolutionary relationships showed similar expression levels. For instance, unigenes comp104335 and comp103272 had the same expression pattern during the development of *P*. *rapae* than comp93707 (Fig 6). One Hsp gene from each family was random selected to validate the expression level using real-time PCR (S5 Table and S2 Fig).

### Differentially expressed immunity-related genes

**Fig 7:**
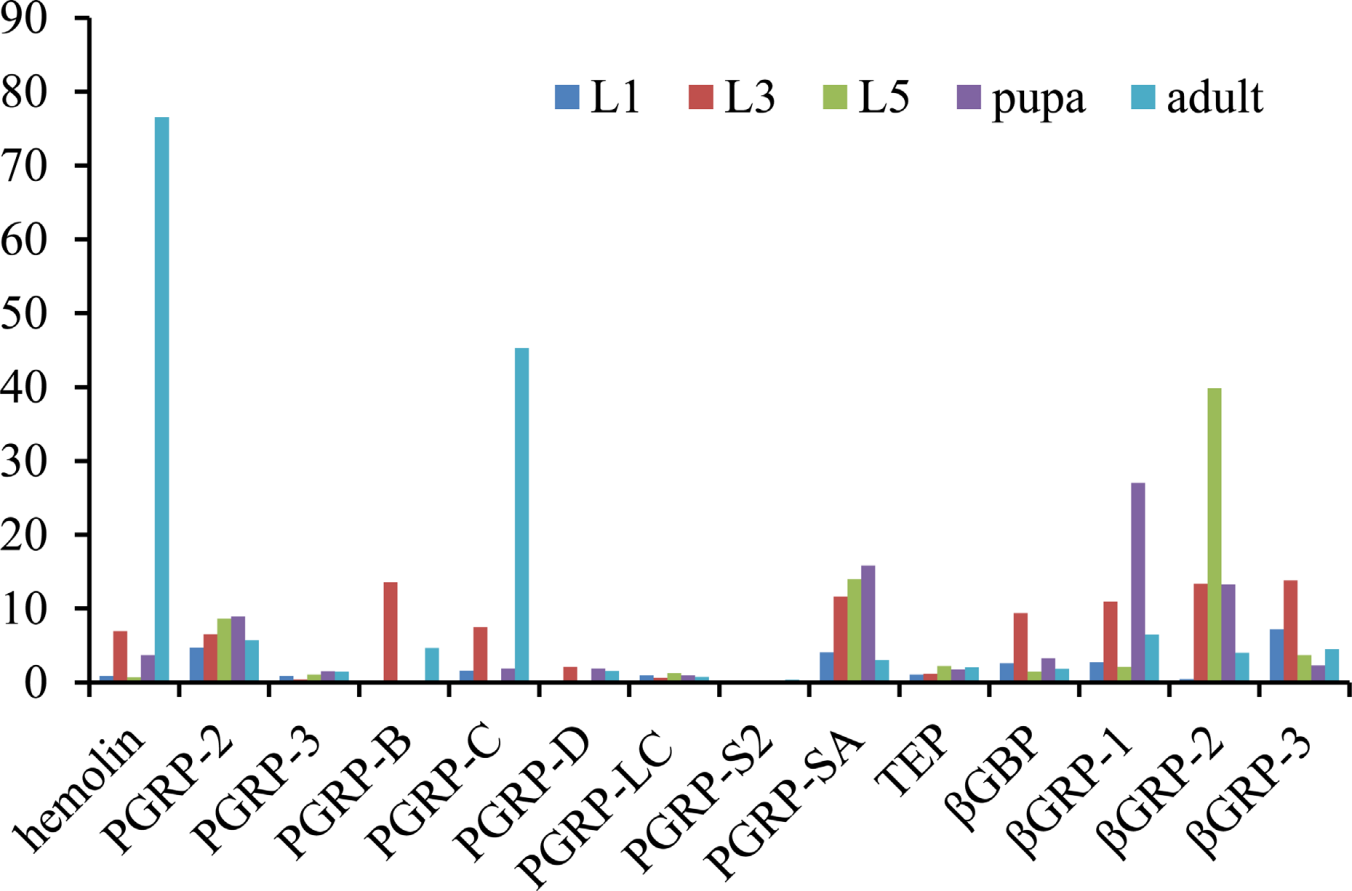
Genes expression of pattern recognition receptors that significantly change in the *Pieris rapae* transcriptome druing development. Expression levels represent the average transformed numbers of RPKM values of genes in the *P*. *rapae* transcriptome.

Within 267 identified immunity related genes, 39 of them were found to be significantly differentially expressed. Most of them did not express in egg stage, except for seven genes, including one beta-1,3-glucan recognition protein genes, one hemolin gene, two prophenoloxidase genes, and two scavenger receptor genes. Two Toll genes found to be expressed after egg stage at relative low levels as well as two toll receptor genes. The key genes *MyD88*, and *IMD* in IMD pathway were not changed expression significantly. However, 35 pattern recognition receptors (PPRs) genes are found to be significantly expressed during the development, including Peptidoglycan recognition proteins (*PPRPs*), *hemolin*, thioester-containing proteins (*TEP*), and β-1,3-glucan recognition proteins (*βGRP*) (Fig 7). *P*. *rapae* Hemolin was extremely up-regulated at the adult stage. During the development of *P*. *rapae*, *βGRP-1*, *βGRP-2*, and *βGRP-3* genes reached the highest expression level at the pupa stage, L5 stage and L3 stages, respectively. Furthermore, different *PGRPs* genes showed various expression levels. *PGRP-2* and *PGRP-SA* genes found to be constituted expressed at a relative higher level during the development compared to *PGRP-3*, *PGRP-D*, *PGRP-LC*, and *PGRP-S2* genes. *PGRP-B* and *PGRP-C* seemed to be expressed impulsely, and they both reached the highest level at the L3 or adult stages.

## Discussion

Although *P*. *rapae* is a major pest of Cruciferae and is involved in the important host-parasitoid model of *P*. *rapae* and *P*. *puparum*, its genetics have not been well studied. In the absence of complete genome sequences, deep transcriptome analysis can provide a basis for performing future gene expression and functional analysis on *P*. *rapae* and can improve our understanding of its biological processes and molecular mechanisms. In the current study, mRNA-seq technology was applied to reveal the developmental transcriptome of *P*. *rapae*. The de novo assembly of short reads without a reference genome remains a challenge with the emergence of bioinformatics tools [27]. Trinity software is widely accepted for its precision [16]. Using the Trinity program, we obtained a total of 330 Mb of sequences, which contained 96,069 unigenes and 238,783 transcripts. They were assembled to dramatically increase the quantity of the genetic data on *P*. *rapae*. The principal metric of an assembly is the length of the transcripts. Depends on approximately 35 Gb of raw data from Illumina sequencing, the 2431 bp of N50 is about three times and four times as length of that of *A*. *lepigone* [11] and *P*. *xylostella* [28], respectively.

The numbers of unigenes annotated in the NR database appear to be similar in three Lepidoptera insects *P*. *rapae*, *P*. *xylostella* [28] and *A*. *lepigone* [11] after *de novo* assembly. The 31,629 unigenes annotated by the NR database showed that the Monarch butterfly (*D*. *plexippus*) shared the highest similarity with the cabbage butterfly (*P*. *rapae*) rather than the silkworm (*B*. *mori*). This similarity may be because *P*. *rapae* is phylogenetically closer to *D*. *plexippus* than to *B*. *mori*, which indicates a common selection of genes between closely related insects. This pattern is different from the transcriptome studies of Lepidoptera *P*. *xylostella* [28] and *A*.*lepigone* [11], as well as Homoptera *Nilaparvata lugens* [29] and *Sogatella furcifera* [30]. They all shared the most similarity with the red flour beetle *Tribolium castaneum*. These patterns are likely due to the insufficient quantity of Lepidoptera transcriptome data published during the study period.

The GO and KEGG annotations allowed us to categorize genes and examine the major metabolic pathways for *P*. *rapae*. The ratio of the number of unigenes annotated by the three main GO categories were roughly equal to another Lepidoptera insect *A*.*lepigone* [11], and was the the highest proportions of each category. It seems that gene products properties are similar in the two Lepidoptera insect. However, we should be cautious with Go annotations. Of the genes with a GO annotation even for *D*. *melanogaster*, no more than 30% have been evaluated by direct assay, expression pattern, genetic interaction, or mutant phenotype [31]. Because the GO annotations for *D*. *melanogaster* were mostly inferred from the computational or indirect evidence, these annotations are generally thought to be of lower quality than those derived from experiments. For other non-model insects, GO annotations were almost inferred from the computational evidence, which may have led to an abundance of false positive. Moreover, based on the KEGG database, both species have over 10,000 genes annotated in approximate 300 canonical pathways. For the seven conserved development-related signaling pathways, such as *Notch* [32], almost all of the *P*. *rapae* orthologs of these key genes were previously unknown. The discovery of these genes may help us elucidate the metabolic mechanism of *P*. *rapae* during development. However, the KEGG annotation is clearly based on orthology-function conjectures, which are known to be false in at least some cases [33]. Therefore, we should pay more attention in future experimental evaluations. Nonetheless, both GO and KEGG can provide us with the overall functional profile of the *P*. *rapae*.

Numerous differentially expressed genes were detected in the pair-wise comparisons of the six developmental stages. Most of the differentially expressed genes were down-regulated in the L1 stage compared to the egg stage, resulting in the largest gene expression differences during the development of *P*. *rapae*. Dramatically changed expression levels between eggs and larvae were also previously found in Lepidoptera *Cnaphalocrocis medinalis* [34]. It may be that during the embryonic development, gene expression is highly dynamic [26]. Cuticular protein genes dramatically change gene expression during development, which provides insight into the relationships among cuticular proteins of various developmental stages. Similar expression patterns were found in *A*. *lepigone* [35] and *Spodoptera litura* [36]. The cuticle, which is an assembly of chitin and cuticle proteins, forms the major part of the skin of arthropods. Frequent molting of insects, may contribute to the variability of the expression levels of cuticular protein genes [37]. These physical properties suggest that cutitular protein is good model to study the function of ecdysteroids and juvenile hormones [38]. Further studies of the differential gene expression between the egg and larvae stages and cuticular protein gene expression may provide more useful information. PRRs are known as key components detecting microorganisms and trigger innate immunity. In current study, we reviewed *PGRPs*, *hemolin*, and *βGRP*. Hemolins have only been found in Lepidoptera, and has not been identified in other insect orders. In *P*. *rapae*, hemolin is a 46.97 kDa protein, which composed of four immunoglobulin domains. The extremely up-regulation at the adult stage, indicating developmentally regulated, which is similar in *M*. *sexta* [39]. The developmental regulation of *hemolin* may activate by the steroid hormone 20-hydroxyecdysone [40]. βGRPs were proteins recognizing and binding the β-1,3-glucan, which is a fungal cell wall component. The highest expression level in the larva or pupa stages, suggests the high risk of *P*. *rapae* in these two developmental stages.*βGRP2* transcripts become highly abundant prior to pupation is a similar with *M*. *sexta* [41]. PGRPs, which were first discovered in Lepidoptera, associate with bacterial peptidoglycans. The various gene expression pattern during the development is all found in *M*. *sexta* [42], *Anopheles gambiae* [43], and *D*. *melanogaster* [44]. A systematic study of P. rapae may reveal their implications in various immune responses.

Heat shock proteins are highly conserved chaperones that respond to environmental changes by facilitating protein folding and preventing protein denaturation. Hsp proteins could be classified into eight families based on homology and molecular weight as follows: sHsp, Hsp10, Hsp40, Hsp60, Hsp70, Hsp90, Hsp100, and Hsp110 [45]. In our previous study [46], the ORFs of three Hsp genes were amplified from *P*. *rapae*, and their expression was confirmed to be changed in response to parasitization by the parasite *P*. *puparum*. In the present transcriptome, six Hsp families including 32 members were obtained, whereas Hsp100 and Hsp110 were not found. Of the 32 Hsp genes, 29 were newly identified and 3 of them were blast hits for previously identified Hsp genes [46]. Considering the development involved in Hsp genes in response to environmental stress [47, 48], the background expression of Hsp genes during development may provide important information for further stress induction. In addition to a stress response, Hsp genes are involved in the development of different organism such as *D*. *melanogaster* [26] and *Liriomyza sativa* [49]. Similar expression levels of Hsp genes closely related in phylogenetic trees during the development of *P*. *rapae*, indicates that genes within a functional group tend to be expressed at similar times [26]. Hsp genes may be involved in similar regulation mechanisms or regulated by the same transcription factors, or may have been acquired through horizontal gene transfer. Previous studies also suggest that the developmental regulation of these hsp genes is controlled, in part, at the level of chromatin structure [50]. A genome wide analysis may confirm this hypothesis after further genome sequencing has been performed.

## Conclusion

A total of 240 million reads were obtained by transcriptome sequencing and the de novo assembly yield 96,069 unigenes with an average length of 1353 nt. Based on similarity searches against databases, 31,629 unigenes were matched to known proteins. The data presented in this study will provide important reference points for further studies of *P*. *rapae* genes and their function. Development-related genes such as cuticular protein, play an indispensable role in the insect cuticle integration, resistance, and innate immunity. If the expression of a key cuticular protein gene inhibited by RNAi technology lead to the death of *P*. *rapae*, this may form the basis of a novel pest control strategy.

## Supporting Information

S1 Fig Histogram presentation of clusters of orthologous groups (COG) classification. The X- axis represents the COG term. The Y-axis shows the number of unigenes.
(TIF)

S2 Fig Expression levels of selected Hsp genes in the *Pieris rapae* during development. Transcript levels for all samples were assessed by real-time PCR. The experiment was perfomred in triplicae (mean ± standard deviation of the mean). All mRNA expression data were normalized to the control gene 18S RNA. None of the selected genes can be detected in the egg stage. Samples from the L1 stage were used as control. The relative expression level to the control using the 2^−ΔΔCT^ method. The significant difference (P<0.5) of each gene expression between other stages and the L1 stage are indicated with asterisks.
(TIF)

S1 File Amino acid sequences of heat shock proteins with entire open reading frames found in *Pieris rapae*. (TXT)

S2 File Amino acid sequences of innate immunity related genes involved in Toll and IMD pathways, and Pattern Recognition Receptors with entire open reading frames in *Pieris rapae*. (TXT)

S3 File The homology relationships of Pieris rapae Hsp10, sHsp, Hsp70, Hsc70, and Hsp90 with their counterparts in other insect species as well as expression levels in P. rapae during development. The trees were generated using the NJ method by Mega5 software. Expression levels were compared with their RPKM values. The used amino acid sequences of other Hsps are from Nv (*Nasonia vitripen*nis), Am (*Apis mellifera*), Bt (*Bombus terrestris*), Cv (*Cotesia vestalis*), Md (*Microplitis demolitor*), Oc (*Oxya chinensis*), Dm (*Drosophila melanogaster*), Tc (*Tribolium castaneum*), Pr (*Pieris rapae*), Px (*Papilio xuthus*), Dp (*Danaus plexippus*), Bm (*Bombyx mori*), Cs (*Chilo suppressalis*), Pv (*Polypedilum vanderplanki*), Fa (*Fopius arisanus*), and Hs (*Harpegnathos saltator*).
(PDF)

S1 Table Summary statistics of illumina sequencing from six developmental stages of *Pieris rapae*. E, L1, L3, L5, L, P, and A refer to the egg, the first stage of larva, the third stage of larva, the fifth stage of larva, pupa, and the adult of *P*. *pieris*, respectively.
(DOC)

S2 Table The Pieris rapae transcriptome involved in KEGG pathways. Partial nodes genes were listed in Table 2.
(XLS)

S3 Table Unigenes hits by key innate immunity related genes involved in Toll and IMD pathways as well as Pattern Recognition Receptors in *Pieris rapae*. (XLSX)

S4 Table List of the stage specific genes expressed during development. (XLS)

S5 Table Primers of Hsp genes used for Real-time PCR. (DOC)
