# Supplementary figures and images for: De Novo Assembly and Developmental Transcriptome Analysis of the Small White Butterfly Pieris rapae
Source: PLoS One. 2016 Jul 18;11(7):e0159258. doi: 10.1371/journal.pone.0159258 (PMC4948883; doi:10.1371/journal.pone.0159258)

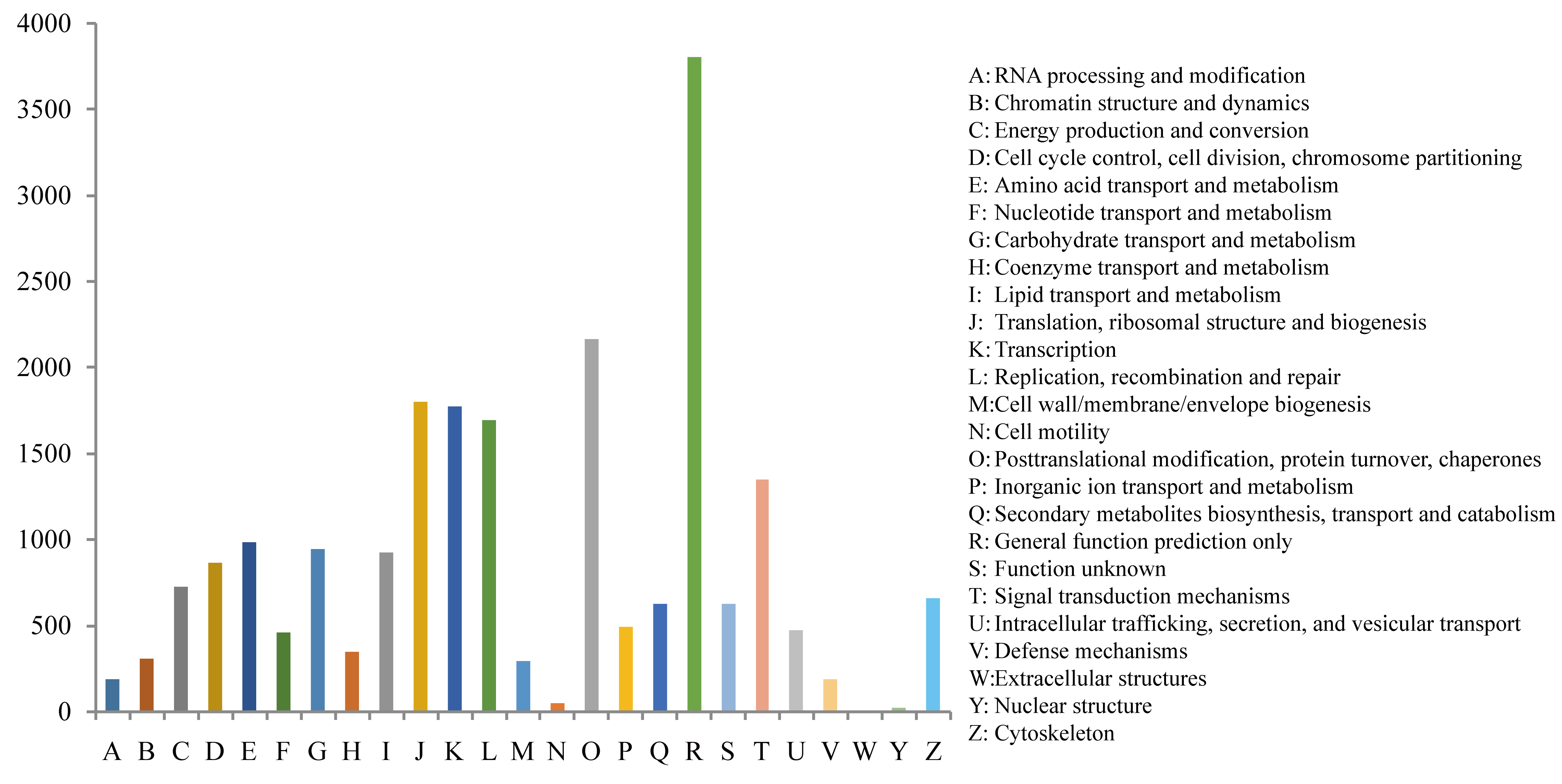

Supplement: S1 Fig — The X- axis represents the COG term. The Y-axis shows the number of unigenes. (TIF) [file pone.0159258.s001.tif]

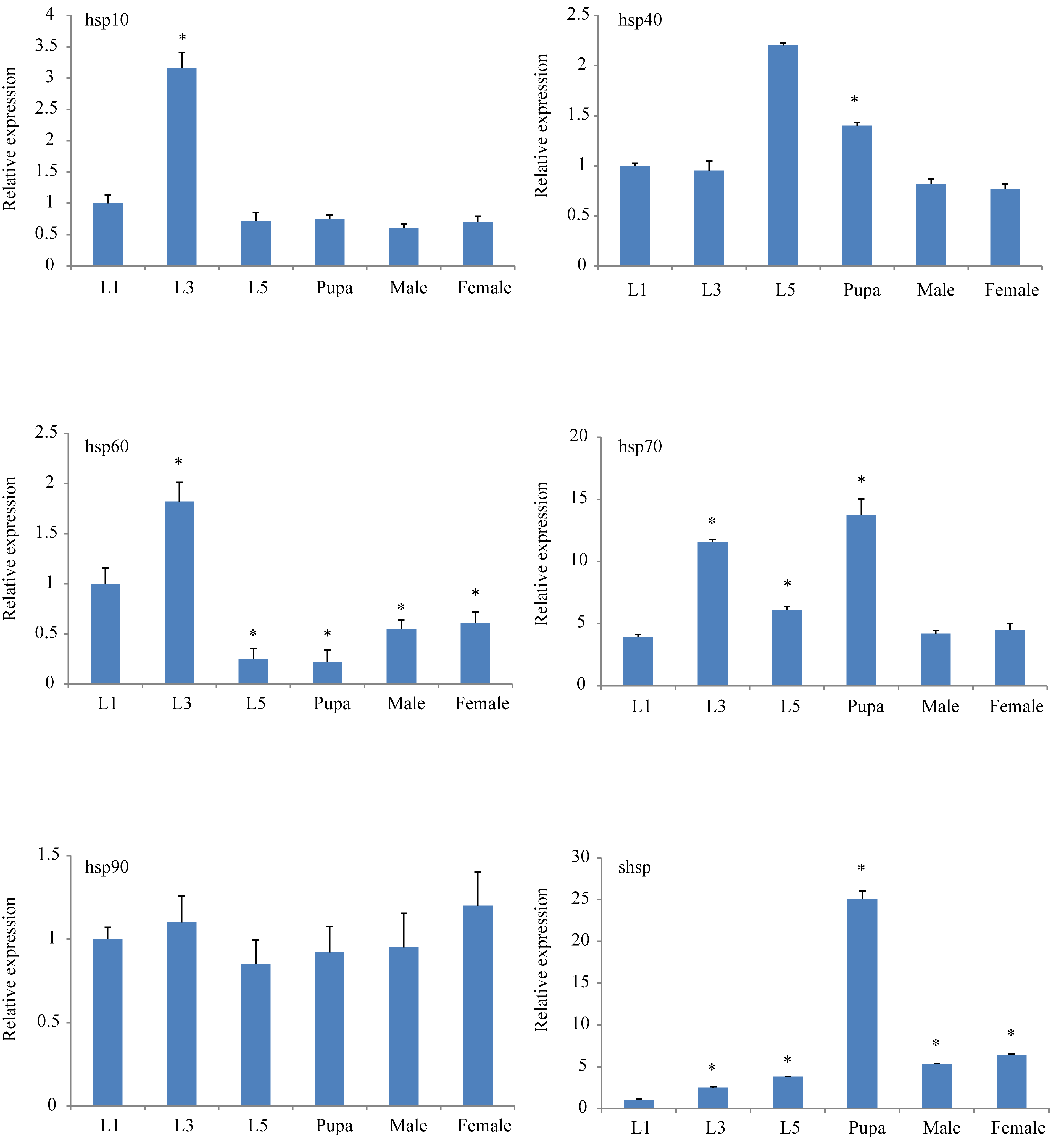

Supplement: S2 Fig — Transcript levels for all samples were assessed by real-time PCR. The experiment was perfomred in triplicae (mean ± standard deviation of the mean). All mRNA expression data were normalized to the control gene 18S RNA. None of the selected genes can be detected in the egg stage. Samples from the L1 stage were used as control. The relative expression level to the control using the 2−ΔΔCT method. The significant difference (P<0.5) of each gene expression between other stages and the L1 stage are indicated with asterisks. (TIF) [file pone.0159258.s002.tif]

hsp10

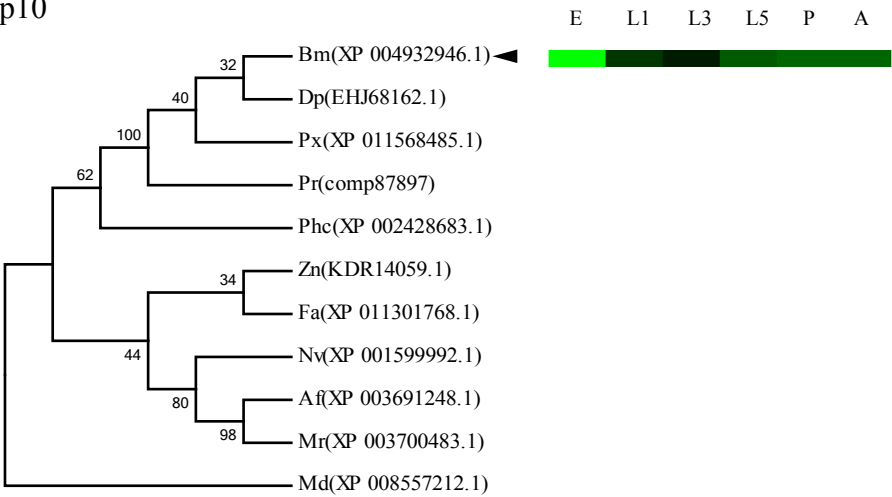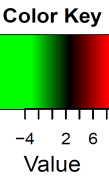

shsp

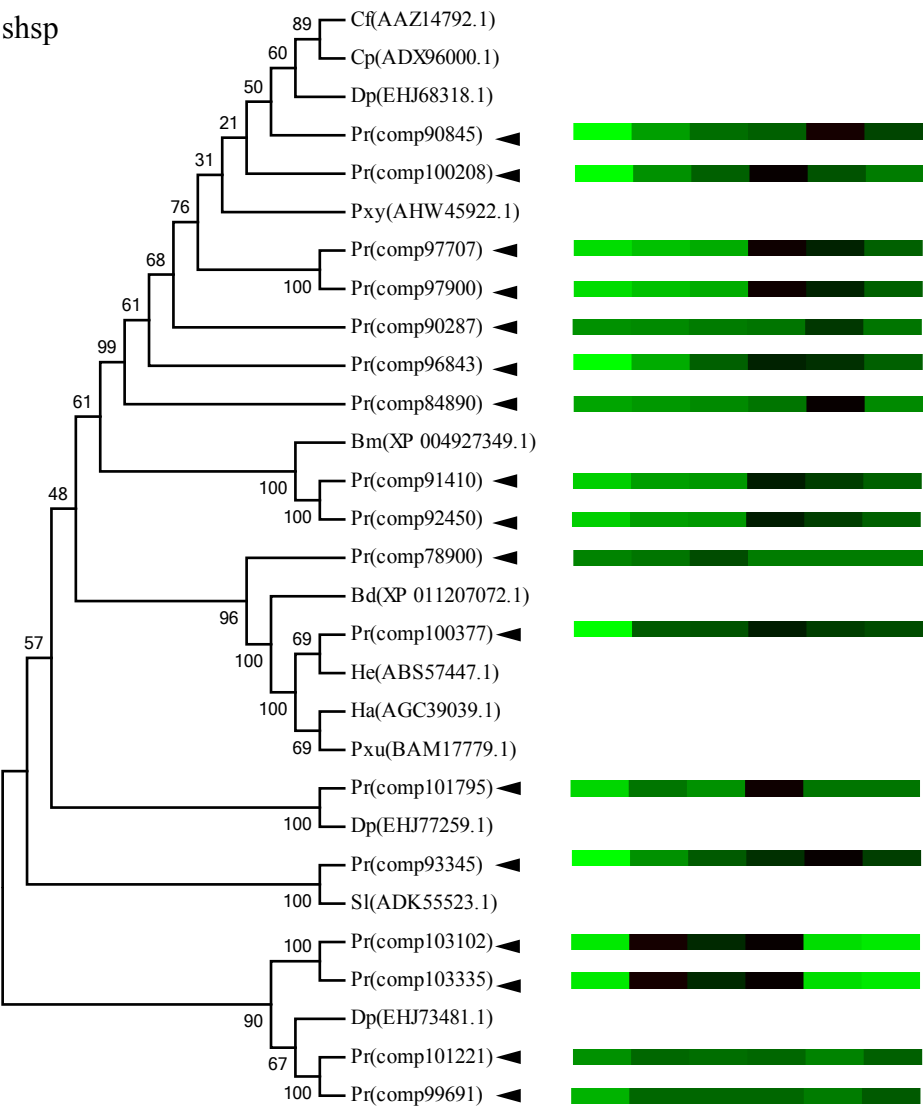

hsc70

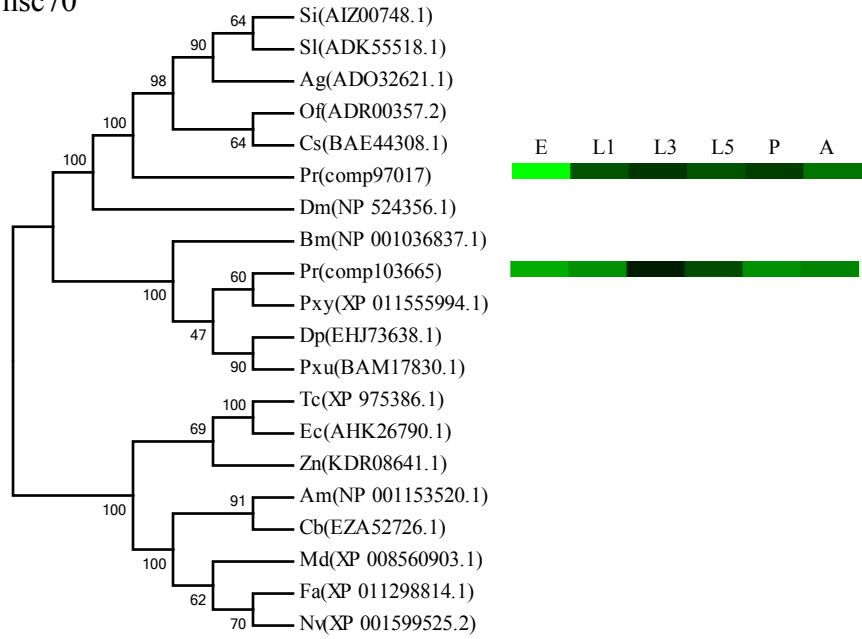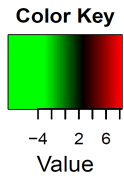

hsp70

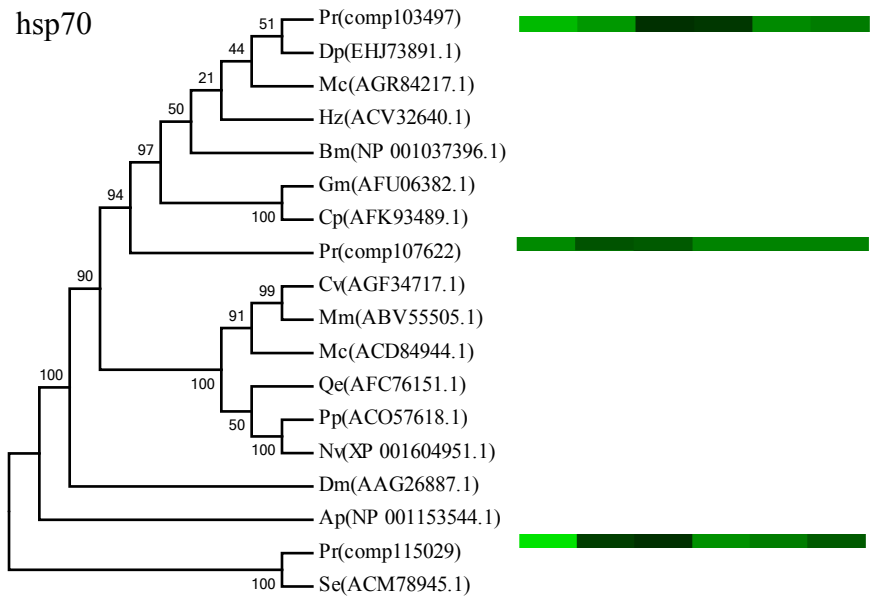

hsp90

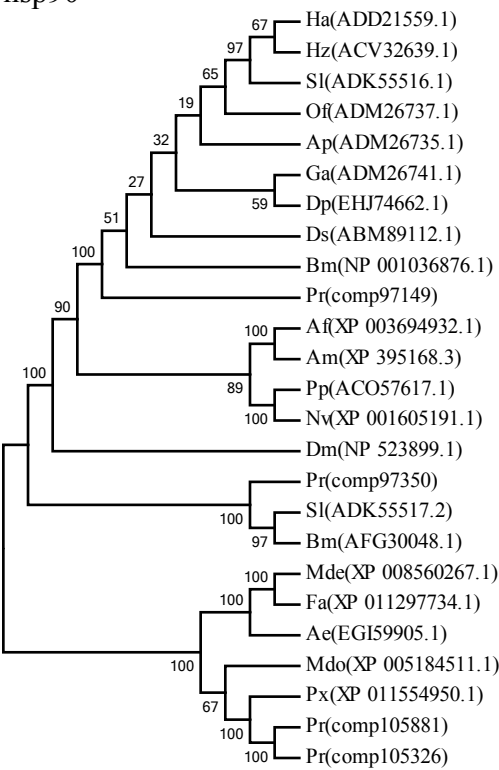

Color Key

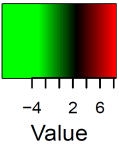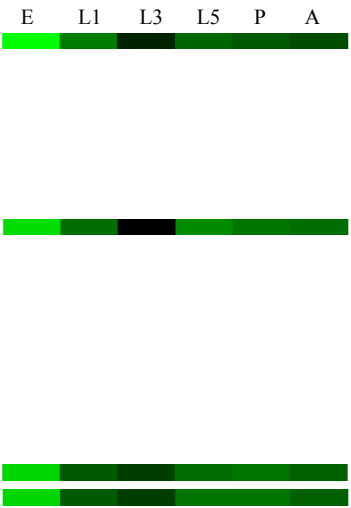

Supplement: S3 File — The trees were generated using the NJ method by Mega5 software. Expression levels were compared with their RPKM values. The used amino acid sequences of other Hsps are from Nv (Nasonia vitripennis), Am (Apis mellifera), Bt (Bombus terrestris), Cv (Cotesia vestalis), Md (Microplitis demolitor), Oc (Oxya chinensis), Dm (Drosophila melanogaster), Tc (Tribolium castaneum), Pr (Pieris rapae), Px (Papilio xuthus), Dp (Danaus plexippus), Bm (Bombyx mori), Cs (Chilo suppressalis), Pv (Polypedilum vanderplanki), Fa (Fopius arisanus), and Hs (Harpegnathos saltator). (PDF) [file pone.0159258.s005.pdf]
