# Supplementary material for: De Novo Assembly and Developmental Transcriptome Analysis of the Small White Butterfly Pieris rapae
Source: PLoS One. 2016 Jul 18;11(7):e0159258. doi: 10.1371/journal.pone.0159258 (PMC4948883; doi:10.1371/journal.pone.0159258)
Supplement: S1 Table — E, L1, L3, L5, L, P, and A refer to the egg, the first stage of larva, the third stage of larva, the fifth stage of larva, pupa, and the adult of P. pieris, respectively. (DOC) [file pone.0159258.s006.doc]

**Table S1. Summary statistics of illumina sequencing from** **six developmental stages of *Pieris rapae***

| Stage | Total number of reads | Total number of bases | Total number of HQ reads | Total number of bases in HQ reads | Percentage of HQ reads |
| --- | --- | --- | --- | --- | --- |
| E | 42,627,274 | 5,328,409,250 | 36,384,698 | 4,548,087,250 | 85.36% |
| L1 | 52,772,096 | 6,596,512,000 | 44,670,160 | 5,583,770,000 | 84.65% |
| L3 | 53,329,586 | 6,666,198,250 | 45,691,942 | 5,711,492,750 | 85.68% |
| L5 | 43,518,160 | 5,439,770,000 | 37,403,300 | 4,675,412,500 | 85.95% |
| P | 43,654,136 | 5,456,767,000 | 37,102,806 | 4,637,850,750 | 84.99% |
| A | 46,335,948 | 5,791,993,500 | 39,632,548 | 4,954,068,500 | 85.53% |
| total | 282,237,200 | 35,279,650,000 | 240,885,454 | 30,110,681,750 | 87.45% |

E, L1, L3, L5, L, P, and A refer to egg, first stage of larva, third stage of larva, fifth stage of larva, pupa, and adults of *P. pieris*, respectively.
