# Supplementary material for: De Novo Assembly and Developmental Transcriptome Analysis of the Small White Butterfly Pieris rapae
Source: PLoS One. 2016 Jul 18;11(7):e0159258. doi: 10.1371/journal.pone.0159258 (PMC4948883; doi:10.1371/journal.pone.0159258)
Supplement: S5 Table — (DOC) [file pone.0159258.s010.doc]

Table S4 Primers of Hsp genes used for Real-time PCR

| Hsp gene | unigene ID | forward primer (5’🡪3’) | reverse primer (5’🡪3’) | product length |
| --- | --- | --- | --- | --- |
| Hsp10 | comp87897_c0 | TCCTCTTTTGGACCGTGTTC | AACTCGGTCGCCTACTTTCA | 184 bp |
| Hsp40 | comp104355_c0 | TTTATGAGCAGCCCAAGAGC | TCCCTTTTGCACTTTCTCGT | 240 bp |
| Hsp60 | comp97942_c0 | GCAAAAGGCACACCAATTTT | GTCACGCCGTCTTTGGTTAT | 189 bp |
| hsp70 | comp103665_c0 | TGCAGAGAAATTCGCAGATG | TCGACCGTCTTCTTTTGCTT | 240 bp |
| hsp90 | comp105881_c0 | CCTGCTGGGAGCTCAGATAC | GTCTAGCTGCAGCCATTTCC | 178 bp |
| sHsp | comp90845_c0 | CGGACAAGGAGAAATTCCAA | CGAGAGAATTGCCGAGAAAC | 151 bp |
| 18S RNA |  | CCAGTGATGGGATGAGTGCT | ACAACCATGGTAGTCGCAGA | 186 bp |
